# Thrombospondin-2 acts as a critical regulator of cartilage regeneration: A review

**DOI:** 10.1097/MD.0000000000033651

**Published:** 2023-04-28

**Authors:** Jing Niu, Yanli Liu, Junjun Wang, Hui Wang, Ying Zhao, Min Zhang

**Affiliations:** a The College of Life Sciences and Medicine, Northwest University, Xi’an, P. R. China; b State Key Laboratory of Military Stomatology & National Clinical Research Center for Oral Disease, Department of General Dentistry and Emergency, School of Stomatology, Fourth Military Medical University, Xi’an, P. R. China; c Department of Anesthesiology and Perioperative Medicine, Xi’an People’s Hospital (Xi’an Fourth Hospital), Northwest University, Xi’an, P. R. China.

**Keywords:** articular cartilage, regeneration, repair, thrombospondin-2 (TSP-2)

## Abstract

The degeneration of articular cartilage tissue is the most common cause of articular cartilage diseases such as osteoarthritis. There are limitations in chondrocyte self-renewal and conventional treatments. During cartilage regeneration and repair, growth factors are typically used to induce cartilage differentiation in stem cells. The role of thrombospondin-2 in cartilage formation has received much attention in recent years. This paper reviews the role of thrombospondin-2 in cartilage regeneration and the important role it plays in protecting cartilage from damage caused by inflammation or trauma and in the regenerative repair of cartilage by binding to different receptors and activating different intracellular signaling pathways. These studies provide new ideas for cartilage repair in clinical settings.

## 1. Introduction

Osteoarthritis (OA) is a chronic autoimmune disease that generally exhibits varying degrees of articular cartilage softening and thinning.^[1]^ Reports show that OA is a musculoskeletal disease with a high prevalence. As the population grows and life expectancy increases, the prevalence of OA is expected to continue to increase globally.^[2]^ An important negative factor in OA is the poor regenerative capacity of cartilage. As a functional tissue located on the surface of bone, articular cartilage typically consists of 4 regions, including a fibrous layer, a proliferative layer, a hypertrophic layer and a calcified cartilage layer, which are characterized by high expression of type II collagen and are rich in proteoglycans. Cartilage plays a crucial role in reducing friction, resisting contact wear and cushioning pressure.^[3]^ However, once the cartilage damage occur under OA, the absence of blood vessels, lymph and nerves in the cartilage tissue and the dense extracellular matrix (ECM) located between the chondrocytes restricts the movement of the chondrocytes, making it difficult for surrounding chondrocytes and nutrients to reach the damaged cartilage area, leaving the defective cartilage tissue unrepaired by the body.^[4]^ Current treatments for cartilage damage include the microfracture method,^[5]^ autologous chondrocyte transplantation,^[6]^ and matrix-induced autologous chondrocyte transplantation.^[7]^ Most of these treatments for cartilage injury only provide short-term relief of symptoms, and no sustainable or effective treatments for regenerative repair of cartilage defects has been seen in the clinic. Therefore, regenerative repair of articular cartilage defects has been a hot topic that has received much attention in clinical treatments. In recent years, tissue engineered cartilage has emerged as a new breakthrough technology that promises to solve this problem by accelerating the repair of cartilage defect areas and providing the possibility of cartilage regeneration.^[8]^

Currently, the chondrogenic induction scheme for seed cells such as mesenchymal stem cells (MSCs) has been widely studied, and many bioactive factors have been shown to promote stem cell differentiation.^[9]^ transforming growth factor-β (TGF-β) is the most important component in the differentiation of adipose-derived stem cells into chondrocytes and in culture. TGF-β binds to the surface receptors of adipose-derived stem cells to form the type I receptor activin receptor-like kinase-5, which promotes Smad2/Smad3 phosphorylation.^[10]^ Insulin-like growth factor-1 is one of the most important regulators of cartilage formation and metabolism, and Insulin-like growth factor-1 increases the expression of COL-II and ACAN in human articular chondrocytes and promotes chondrocyte Sox9 expression and COL2α1 formation.^[11]^ Sacramento et al^[12]^ found that after bone morphogenetic protein induction, a large number of bone mesenchymal stem cells (BMSCs) expressed alkaline phosphatase. The expression level became more pronounced as the induction time increased, indicating that the number of BMSCs that differentiated into chondrocytes increased, showing that bone morphogenetic protein can induce BMSCs to differentiate into chondrocytes. In addition, parathyroid hormone plays a role in regulating bone remodeling and controlling calcium homeostasis, and its enhanced effect on cartilage differentiation and the inhibition of hypertrophy has made it a hot topic in recent years in the study of chondrogenic differentiation of BMSCs.^[13]^ An increasing number of bioactive factors have been shown to promote the differentiation of stem cells and facilitate tissue regeneration, but many of them remain to be fully explored. The role of thrombospondin-2 (TSP-2) in the development of cartilage regeneration has been investigated in recent years. The protein encoded by this gene belongs to the TSP family.^[14]^ It is a disulfide-linked homotrimeric glycoprotein that mediates cell–cell and cell-matrix interactions. This protein has been shown to be a potent inhibitor of tumor growth and angiogenesis.^[15]^ Furthermore, it has been shown that TSP-2 in hUB-MSCs promotes the differentiation of chondroprogenitor cells to chondrocytes through paracrine effects.^[16]^ After searching PubMed and web of science for relevant research articles from 1991 to 2022 using the keyword TSP-2 in combination with cartilage, we reviewed recent advances in the use of TSP-2 for cartilage regeneration and the mechanism of action, which is expected to provide new targets for clinical cartilage repair. The search included primary research, reviews, clinical trials and original articles. Gray literature and non-English articles were excluded from the analysis.

## 2. Structure and function of TSP-2

### 2.1. Structure of TSP-2

**Figure 1.**
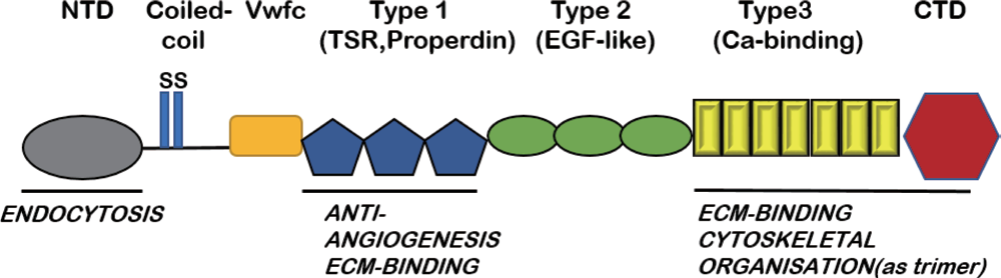
Structural diagram of the TSP-2 protein. TSP-2 = thrombospondin-2.

As an important member of the thrombospondin family, TSP-2 was first identified in 1991 by Bornstein et al^[17]^ in the mouse fibroblast cell line NIH 3T3, and it was later shown to be an ECM glycoprotein that was widely distributed in epithelium-derived tissues. TSP-2 consists of an N-terminal heparin-binding domain, a precollagen homology domain, 3 type I repeats, 3 epidermal growth factor-like type II repeats, 7 calcium-binding type III repeats and a C-terminal globular structural domain (Fig. 1).^[18,19]^ TSP-2 can regulate cell proliferation, adhesion and apoptosis by binding to cell surface receptors (e.g., integrins), ECM components (e.g., core proteoglycans, proteoglycans), enzymes (e.g., matrix metalloproteinases) and calcium ions.^[20]^ In addition to TSP-2, platelet response protein family members include TSP-1, TSP-3, TSP-4, and TSP-5. Among them, TSP-2 and TSP-1 are homologs, and the 2 genes encode similar protein structures, but there are some differences in gene and protein expression and the regulation of cells.^[21]^

### 2.2. Function of TSP-2

**Figure 2.**
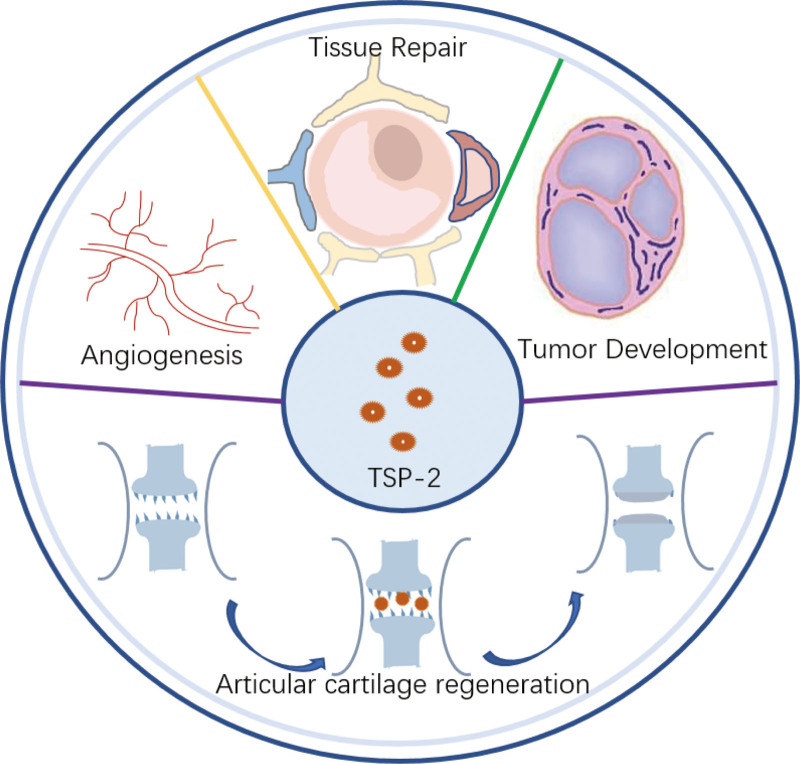
Diagram showing how TSP-2 plays a role in tissue. TSP-2 = thrombospondin-2.

TSP-2 expression is low in normal adult tissues, and its distribution is essentially the same as during development; this factor is mainly present in the dermis, cartilage, bone and blood vessels.^[22–24]^ TSP-2-null mouse skin fibroblasts exhibit significant differences in the expression of adhesion and multiple matrix proteins (including fibronectin) and diffusion, and astrocyte-derived TSP-2 is critical for maintaining physiological levels of MMP-2 and MMP-9 during the foreign body response in the brain and contributes to blood–brain barrier repair.^[25]^ During mammalian development, TSP-2 is synthesized mainly in connective tissues. Knockdown of TSP-2 leads to skin fragility, tendon and ligament laxity, and increased vascular density in the dermis, adipose tissue, and thymus in mice. Lange-Asschenfeldt et al^[26]^ induced a delayed hypersensitivity response by local sensitization of ear skin in wild-type and TSP-2-deficient mice and found that TSP-2 expression was upregulated in the inflamed skin of wild-type mice, mainly in dermal fibroblasts and microvessels. TSP-2 deficiency significantly enhanced the inflammatory response, which was accompanied by angiogenesis, edema formation, and increased inflammatory infiltration. These results suggest a role for TSP-2 in inhibiting inflammation and angiogenesis, but it has also been shown that TSP-2 does not play an important role in adipose tissue-associated angiogenesis or adipogenesis.^[27]^ The TSP-2 and TSP-1 genes show a high degree of sequence homology. Interestingly, TSP-2 has a stronger inhibitory effect on tumor growth and angiogenesis than TSP-1.^[28]^ Indeed, several researchers have reported that TSP-2 is a negative regulator of cancer-associated angiogenesis and tumor aggressiveness in a variety of cancers. Nakamura et al^[29]^ showed that TSP-2 expression was negatively correlated with cancer cell proliferation and MMP-9 expression. The clinical relevance of TSP-2 in many different cancers has also been explored. TSP-2 mediates integrin production to mediate matrix metalloproteinase-13 (MMP-13) expression, invasion and migration in lung cancer cells.^[30]^ TSP-2 also inhibits cell invasion by downregulating MMP-9 and uridylyl phosphate adenosine activity in pancreatic cancer cell lines, suggesting that TSP-2 may be a potent inhibitor of pancreatic cancer metastasis.^[31]^ In addition, the effect of TSP-2 on angiogenesis may also indirectly affect the tumor immune response, which is a topic worthy of further investigation (Fig. 2).

### 2.3. The role of TSP-2 in cartilage development and regeneration

In addition to blood vessels, tumors and skin, TSP-2 is closely associated with the development of bone and cartilage tissue. TSP-2-deficient mice have increased bone formation, and bone geometry is altered, as evidenced by an increase in cortical bone density and a reduction in the bone marrow area. TSP-2 is highly expressed in the articular chondrogenic regions of fetal and adult mice.^[22]^ Kyriakides et al^[32]^ disrupted the Thbs2 gene through homologous recombination in embryonic stem cells and bred TSP-2-null mice by blastocyst injection and appropriate breeding of mutant animals. Histological examination and pQCT analysis of the tibias and femurs of wild-type and mutant mice revealed increased total bone density and cortical thickness. TSP-2 was highly expressed in intramembranous and endochondral bone formation sites in developing bones. Nishiwaki et al^[33]^ showed that TSP-2 expression in osteoblasts was regulated by the activator protein-1 transcription factor transcription factor activator protein-1. In adult bone, the expression level of TSP-2 was relatively low but increased significantly with cartilage damage. This finding is consistent with other studies demonstrating an increase in TSP-2 during skin, muscle and heart tissue injury. These findings may reflect a role for TSP-2 in the development of cartilage regeneration (Fig. 2). Fracture model studies showed that TSP-2-knockout mice had up to 30% higher osteogenesis and 40% lower cartilage volume than wild-type mice,^[16]^ which suggests that TSP-2 may be involved in cartilage formation and cartilage defect repair.

#### 2.3.1. The role of TSP-2 in mesenchymal stem cell differentiation

MSCs possess powerful immunomodulatory and anti-inflammatory functions, which are mainly mediated by secreted proteins, and contribute to cartilage tissue repair. Previously, the use of MSCs for cartilage tissue repair was thought to be based on their direct differentiation into chondrocytes to regenerate cartilage or by stimulating endogenous chondrocytes to repair cartilage, but there is now increasing evidence that the cartilage repair capacity of MSCs is mediated by their paracrine effects. MSCs can secrete a variety of growth factors and trophic factors, promote their own differentiation into chondrocytes, promote the proliferation and growth of resident chondrocytes, and secrete anti-inflammatory and immunomodulatory factors to regulate the microenvironment of damaged tissues, which can promote cartilage tissue regeneration.^[34–38]^ It is well known that the proliferation of remaining host synovial chondrocytes and chondrocyte differentiation from transplanted stem cells are key factors in OA cartilage repair, and among the various growth factors, transforming growth factor-β, insulin-like growth factor and fibroblast growth factor (FGF) are potent regulators of chondrocyte proliferation and differentiation.^[39,40]^ Notably, TSP-2 was recently shown to contribute to the chondrogenic potential of human umbilical cord blood MSCs (hUCB-MSCs) in cartilage defects.^[22]^ Hankenson et al^[41]^ reported that TSP-2 was not expressed in hematopoietic lineage cells, whereas bone marrow MSCs were the main source of TSP-2. Jeong et al^[42]^ found considerable differences in the chondrogenic differentiation potential of hUCB-MSCs, Human bone marrow-derived MSCs and human adipose tissue-derived MSCs obtained from different donors, which was influenced by TSP-2; therefore, the expression level of TSP-2 in bone marrow MSCs could be used as an indicator for optimal bone marrow MSC selection to promote effective cartilage regeneration. hUCB-MSCs can stimulate the differentiation of local endogenous chondrogenic progenitor cells through TSP-2, ultimately leading to cartilage regeneration. MSC-based therapies have shown variable efficacy in the treatment of various diseases, including cartilage defects. The chondrogenic differentiation potential of MSCs is influenced by TSP-2 levels in differentiated cells. Therefore, TSP-2 levels can be used as a marker for selecting MSCs with superior chondrogenic differentiation potential for cartilage regeneration therapy (Table 1).

**Table 1:** TSP-2 plays a role in cartilage regeneration and repair.

	Cell type/tissue	Main functions	References
TSP-2 plays a role in cartilage formation	Cartilage	TSP-2 is highly expressed in cartilage tissue	Kyriakides TR, et al^[22]^ (1998)
	hUCB-MSCs	TSP-2 affects chondrogenic differentiation potential	Jeong SY, et al^[42]^ (2015)
	Osteoprogenitor cells	TSP-2 affects abnormalities in collagen fibers and increased ligamentous laxity.	Hankenson KD, et al^[41]^ (2000)
The role of TSP-2 in cartilage regeneration and repair	hUCB-MSCs/Cartilage	TSP-2 expression is elevated in OA	Jeong SY, et al^[16]^ (2013)
			Han J, et al^[43]^ (2022)
	Cartilage and bone	TSP-2 affects the ratio of bone to cartilage formation	Taylor DK, et al^[44]^ (2009)
			Alford AI, et al^[45]^ (2012)
	hUCB-MSCs	rhTSP-2 supplementation can affect the chondrogenic differentiation	Jeong SY, et al^[42]^ (2015)
	hADMSCs	TSP-2 and hADMSCs have synergistic effects on cartilage regeneration.	Shin K, et al^[46]^ (2019)

hUCB-MSCs = human umbilical cord blood MSCs, hADMSCs = human adipose-derived MSCs, OA = osteoarthritis, TSP-2 = thrombospondin-2.

#### 2.3.2. The role of TSP-2 in cartilage regeneration and repair

TSP-2 is closely associated with the development and regeneration of cartilage. Interestingly, the expression of TSP-2 is increased in OA, and its expression level correlates with disease severity. Treatment with synovial fluid (SF) obtained from patients with OA increased this paracrine effect of hUCB-MSCs on cartilage progenitor cells compared to that in the untreated group. Jeong SY analyzed the secretome of OA SF-stimulated hUCB-MSCs using a biotin-labeled antibody-based array. TSP-2 specificity was increased in OA SF-treated hUCB-MSCs. To further determine the role of TSP-2, exogenous TSP-2 was added to chondroprogenitor cells in microculture and revealed that TSP-2 induced chondrogenic effects on chondroprogenitor cells.^[16,43]^ A study by Taylor et al^[44]^ showed that TSP-2-deficient mice had reduced expression of COL-II and Sox9 (markers of chondrocyte differentiation) but increased expression of osteocalcin (a marker of osteoblast differentiation). Chondrogenesis was restored in TSP-2-deficient mice through the expression of TSP-2 using an adenovirus 3 days after fracture.^[45]^ This finding suggests the importance of TSP-2 in controlling the cartilage-to-bone ratio during fracture healing to accelerate healing or promote cartilage regeneration. It has also been shown that the expression level of TSP-2 affects the differentiation potential of cartilage progenitor cells in embryonic and adult mice. This finding is equally indicative, showing that siRNA-mediated TSP-2 knockdown and supplementation with recombinant TSP-2 can affect cartilage differentiation in hUCB-MSCs through many signaling pathways (Table 1).^[42]^

In addition, TSP-2 plays a role in cartilage hypertrophy, and knockdown of TSP-2 significantly increases RUNX2 and MMP-13 levels in the cartilage microspheres of hUCB-MSCs during induction of cartilage hypertrophy; in contrast, the addition of rhTSP-2 reduces RUNX2 and MMP-13 levels to levels comparable to those of the controls.^[42]^ These data suggest that TSP-2 attenuates the expression of hypertrophy-related markers in hUCB-MSCs cultured under hypertrophy-inducing conditions. The pathology of OA is characterized by degradation of the ECM, which results from an increase in matrix metalloproteinases, aggregated proteases and proinflammatory cytokines, and MMP-13 typically degrades type II collagen in the cartilage ECM.^[47]^ The decrease in MMP-13 due to TSP-2 may play a role in maintaining cartilage matrix homeostasis. These studies suggest that TSP-2 not only maintains the chondrocyte phenotype but also protects the cartilage matrix from degradation. In male New Zealand White rabbits undergoing ACL transection surgery, TSP-2 was injected intra-articularly 8 weeks later, and in an OA model, TSP-2 treatment increased the expression of cartilage formation markers (SOX9 and type II collagen), while TSP-2 siRNA treatment inhibited the expression of these factors. In vivo studies in animals also showed that the combination of human adipose-derived MSCs (hADMSCs) and TSP-2 further induced a lower degree of cartilage degeneration, bone fragmentation and ECM loss than hADMSCs or TSP-2 alone. In addition, the combination treatment resulted in a significant decrease in synovial inflammatory cytokines, especially tumor necrosis factor-α, and a significant increase in articular cartilage regeneration. This finding indicates that TSP-2 and hADMSCs have synergistic effects on cartilage regeneration (Table 1).^[46]^

#### 2.3.3. TSP-2-related molecular regulation in chondrogenesis

In recent years, signaling pathways that are closely related to cartilage growth and development have been investigated, and various signaling stimuli from internal and external sources can converge on SOX9, which is the main transcription factor of growth plate chondrocytes, through a complex signaling cascade to ensure the normal growth and development of the cartilage system.^[48]^ Several signaling pathways have been shown to regulate the growth and development of growth plate cartilage, and some studies have been conducted on the mechanism of TSP-2 in cartilage regeneration.

**Figure 3.**
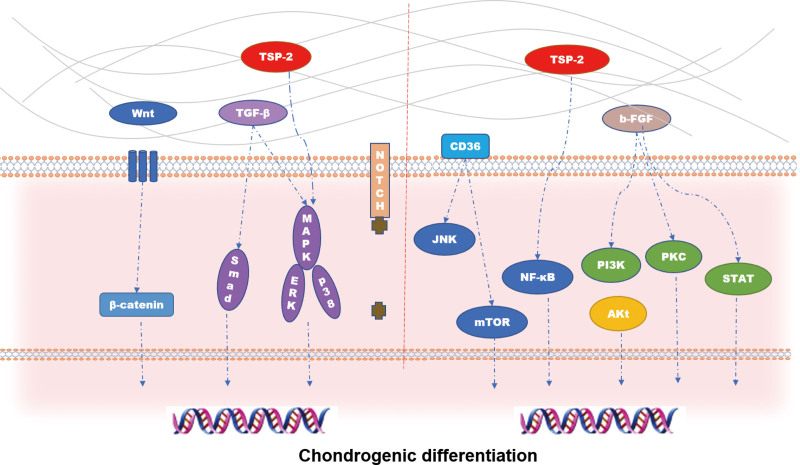
Signaling pathways involved in the regulation of TSP-2 in cartilage. TSP-2 = thrombospondin-2.

The Wnt signaling pathway plays an important role in embryonic development, cell growth and stem cell proliferation; in particular, it is important in balancing the complex spatiotemporal interactions between bone remodeling and cartilage degeneration.^[49–53]^ It was shown that TSP-2 could inhibit the expression of the hypertrophy-related genes RUNX2 and MMP-13, and the inhibition of hypertrophy-related genes by TSP-2 was mediated through the Wnt/β-catenin signaling pathway, as assessed by measuring (p) GSK-3β, β-catenin levels in the medium. This result suggests that TSP-2 can act through Wnt/β-catenin. Mitogen-activated protein kinase is one of the conserved signal transduction systems in cartilage and plays a crucial role in cartilage differentiation.^[53–55]^ Shin et al^[46]^ showed that TSP-2 regulates chondrogenic differentiation in hADMSCs, which is mediated by the Notch signaling pathway. It has also been shown that the Notch signaling pathway regulates chondrocyte hypertrophy by regulating Sox9 expression.^[56]^ However, it was previously shown that in a mouse model with enhanced Notch signaling, Notch signaling acts upstream of Sox9 and Runx2 to inhibit chondrocyte proliferation.^[57]^ Notch negatively regulates chondrocyte differentiation in the mesial bone by suppressing Sox9 expression.^[58]^ The deletion of Notch receptors in osteochondral progenitor cells promotes chondrocyte differentiation.^[59]^ TGF-β is an important cytokine that maintains chondrocyte phenotype and is one of the necessary conditions for the induction of chondrogenic differentiation in BMSCs.^[60]^ The TGF-β/Smad signaling pathway is the main signaling pathway by which TGF-β exerts its effect and is closely related to cartilage development.^[61]^ According to the structural prediction model of TSP-2 and the literature, TSP-2 can interact with TGF-β, which can further activate the PKC, mitogen-activated protein kinase, and Akt/mTOR signaling pathways and is involved in the chondrogenic differentiation of BMSCs.^[62,63]^ In addition, TSP-2 can bind to many cytokines and receptors due to its unique structure and can mediate various signaling pathways. CD36 is currently known to be a pattern recognition receptor, and TSP-2 can act through CD36,^[64]^ which can activate JNK1/2 kinase and release Interleuking 6.^[65]^ Hou et al^[66]^ detected the induction of nuclear factor kappa-B (NF-κB) promoter activity by TSP-2 using luciferase reporter assays and confirmed that the transcriptional activation of NF-κB was stimulated by TSP-2. And it has been demonstrated that NF-κB can regulate the expression of Sox9.^[67]^ In addition, the structure of TSP-2 suggests that b-FGF is likely to bind to TSP-2; thus, b-FGF is likely to be an upstream signaling molecule by which TSP-2 promotes chondrogenic differentiation (Fig. 3) (Table 2).^[63,68]^

**Table 2:** TSP-2-related molecular regulation in chondrogenesis.

Cell type	Response/regulation	Cell signaling	References
hUCB-MSCs	Runx2 and MMP-13	Wnt/β-catenin	Jeong SY, etal^[42]^ (2015)
			Nusse R, etal^[49]^ (2017)
			Monteagudo S, etal^[50]^ (2017)
			Usami Y, etal^[51]^ (2016)
			Takahata Y, etal^[52]^ (2022)
			Sassi N, et al^[53]^ (2014)
hADMSCs	Sox9 and Runx2	Notch	ShinK, etal^[46]^ (2019)
			Wang C, etal^[56]^ (2016)
			Chen S, et al^[57]^ (2013)
BMSCs	Smad	TGF-β	Samsa WE, etal^[60]^ (2017)
			Wang C, etal^[61]^ (2020)
Endothelial cell	IL-6	JNK	Koch M, etal^[64]^ (2011)
			Geng J, etal^[65]^ (2018)
Limb bud cells	Sox9	NF-κB	Hou CH, etal^[66]^ (2021)
			Guo M, etal^[67]^ (2015)
Chondrocytes	Acan and CoI2A1	FGF	Horiguchi H, etal^[63]^ (2004)
			Hayes AJ, etal^[63]^ (2022)

BMSCs = bone mesenchymal stem cells, FGF = fibroblast growth factor, hADMSCs = human adipose-derived MSCs, hUCB-MSCs = human umbilical cord blood MSCs, IL-6 = interleuking 6, MMP-13 = matrixmetalloproteinase-13, NF-κB = nuclear factor kappa-B, TGF-β = transforming growth factor-β.

## 3. Conclusion and perspectives

In the past decades, there have been many studies on various factors affecting the chondrogenic differentiation of bone marrow MSCs, and many factors have been shown to promote the chondrogenic differentiation of bone marrow MSCs. With increasing research on MSCs, MSC-based regeneration therapies have shown the advantages of repairing cartilage damage in OA, and research on MSC-based tissue engineered cartilage has advanced. Existing studies have shown that TSP-2, which is an ECM protein, may have an effect on the chondrogenic differentiation of bone marrow MSCs. Experiments have been performed to investigate the important role of TSP-2 in cartilage regeneration, and some progress has been made. TSP-2 has been shown to contribute to cartilage repair and OA treatment through in vitro or animal studies. Topical injection of TSP-2 is simple to perform and has shown satisfactory results in cartilage repair. In addition, TSP-2 can be used together with MSCs to exert synergistic effects on cartilage regeneration. These advances make the use of TSP-2 safer and more reliable and may contribute to the application of TSP-2 in the clinical setting. These properties of TSP-2 and the aforementioned biological potential of cartilage lead to the possibility of using this protein in joint repair, suggesting a role in the prevention and treatment of articular cartilage diseases associated with aging and metabolic defects. This potential of TSP-2 has generated interest and promises to offer new hope for articular cartilage repair therapy.

## Acknowledgements

This research was supported by the National Natural Science Foundation of China (No. 31971248, 81901052), the National Clinical Research Center for Diseases Project (LCA202007) and Shanxi Science and Technology Innovation Team Project (2021TD-46).

## Author contributions

**Conceptualization:** Junjun Wang, Min Zhang.

**Data curation:** Jing Niu, Yanli Liu.

**Funding acquisition:** Min Zhang.

**Resources:** Jing Niu, Yanli Liu, Junjun Wang, Hui Wang, Ying Zhao.

**Writing – original draft:** Jing Niu, Yanli Liu, Junjun Wang.

**Writing – review & editing:** Min Zhang.
